# Cervical Anastomotic Leakage After Minimally Invasive McKeown Esophagectomy in the SAFER Surgical System: Clinical Course and Predictors of Delayed Healing

**DOI:** 10.1111/1759-7714.70314

**Published:** 2026-06-10

**Authors:** Youyu Zhang, Weishi Wang, Yongjun Yu, Lixia Yin, Jintao Li, Weixin Liu, Xuefeng Zhang, Xingbo Zhang, Hong Yang, Youyu Wang, Shenhai Wei, Yong Li

**Affiliations:** ^1^ Department of Thoracic Surgery Beijing Huaxin Hospital (The First Hospital of Tsinghua University) Beijing China; ^2^ Department of Thoracic and Cardiovascular Surgery Zhuzhou Central Hospital Zhuzhou Hunan China; ^3^ Department of Cardiothoracic Surgery The Affiliated Hospital of Southwest Medical University Luzhou Sichuan China; ^4^ Department of Cardiovascular Thoracic Surgery The Second Hospital of Chifeng Chifeng Inner Mongolia Autonomous Region China; ^5^ Department of Oncology National Cancer Center/National Clinical Research Center for Cancer/Cancer Hospital, Chinese Academy of Medical Sciences and Peking Union Medical College Beijing China; ^6^ Department of Thoracic Surgery Beijing Dawang Road Emergency Rescue Hospital Beijing China; ^7^ Department of Thoracic Surgery The First Affiliated Hospital of Dalian Medical University Dalian Liaoning China; ^8^ Department of Thoracic Surgery Sun Yat‐Sen University Cancer Center; State Key Laboratory of Oncology in South China; Guangdong Provincial Clinical Research Center for Cancer Guangzhou Guangdong China; ^9^ Department of Thoracic Surgery Sichuan Provincial People's Hospital, University of Electronic Science and Technology of China Chengdu Sichuan China

**Keywords:** cervical anastomotic leakage, esophageal cancer, posterior mediastinal reconstruction, retrosternal reconstruction, SAFER surgical system

## Abstract

**Background:**

Cervical anastomotic leakage (AL) remains a major complication after esophagectomy. We investigated the clinical course of cervical AL after minimally invasive McKeown esophagectomy within the SAFER surgical system and identified predictors of delayed healing.

**Methods:**

This retrospective study included 517 consecutive patients who underwent minimally invasive McKeown esophagectomy with retrosternal reconstruction under SAFER between January 2023 and December 2025. Among them, 50 developed cervical AL. A posterior mediastinal comparator cohort of 37 patients with cervical AL was included for comparison of leak severity and healing outcomes. AL severity was assessed using ECCG and Clavien‐Dindo classifications. Cox regression was used to identify predictors of time to closure.

**Results:**

In SAFER group, cervical AL occurred in 50 of 517 patients (9.7%). Compared with the historical same‐surgeon PM leakage cohort, SAFER related leaks showed a lower severity profile, with fewer advanced ECCG events and a less severe Clavien‐Dindo distribution. All SAFER group patients with cervical AL achieved eventual healing, and the median time to closure was shorter in the SAFER group. Low postoperative day 1 serum albumin (< 35 g/L) independently predicted delayed healing. Patients with low albumin had longer healing time and longer postoperative hospitalization.

**Conclusions:**

Within the SAFER surgical system, cervical AL after minimally invasive McKeown esophagectomy was predominantly low grade, manageable, and ultimately healed in all affected patients. Postoperative day 1 serum albumin may help identify patients at risk of prolonged recovery.

## Graphical abstract

In a 517‐patient cohort, the SAFER surgical system was associated with predominantly low‐grade, manageable cervical anastomotic leakage after minimally invasive McKeown esophagectomy, with no ECCG grade III leakage or leak‐related mortality. POD1 serum albumin helped identify patients at risk of delayed healing.

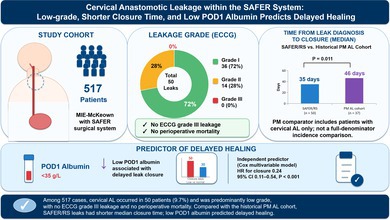

## 1 Introduction

Esophageal cancer remains a major global health burden. In 2022, approximately 511 000 new cases and 445 000 deaths were attributed to this disease worldwide, and curative resection remains a cornerstone of treatment for resectable disease [1, 2]. Yet esophagectomy is technically demanding and associated with substantial postoperative morbidity. Anastomotic leakage (AL) is among its most feared complications because it prolongs hospitalization, increases the need for reintervention, and worsens short‐term outcomes [3, 4, 5]. For this reason, AL has become a central quality metric in esophageal surgery.

Digestive tract reconstruction after esophagectomy is usually performed through either the posterior mediastinal (PM) or retrosternal (RS) route. The superiority of one route over the other remains unsettled. A recent meta‐analysis suggested that the PM route may be associated with a lower leak rate [6], whereas Japanese national data and other retrospective series have shown route‐specific trade‐offs, especially with respect to leakage, wound infection, and pulmonary morbidity [7, 8]. Contemporary Chinese survey data indicate that reconstruction through the esophageal bed remains dominant, implying that retrosternal reconstruction has not yet been broadly standardized in China [9].

When a cervical leak occurs, however, retrosternal reconstruction may offer distinctive anatomical and management advantages. The anastomosis is relatively superficial, wound care and drainage are more straightforward, and the infectious source is relatively separated from the thoracic cavity [5, 8, 10]. These features may contain the clinical consequences of leakage even if they do not abolish leakage itself. At the same time, retrosternal reconstruction is not intrinsically low risk. Several studies have shown that thoracic inlet narrowing, conduit compression, and adverse anastomotic geometry may increase leak risk in this setting [11, 12, 13, 14]. Therefore, the clinical performance of the retrosternal route depends heavily on technical refinement and pathway standardization.

Based on these considerations, our team, in collaboration with multiple centers in China, gradually developed and refined the SAFER (Secure Approach for Esophagectomy with Retrosternal Reconstruction) surgical system. SAFER is an optimized surgical system for esophageal cancer centered on the retrosternal reconstruction route. It was developed to address postoperative complications, particularly anastomotic leakage and the potentially fatal infections that may follow, with the aims of reducing surgical risk and improving surgical quality. Importantly, SAFER should not be regarded as merely a reconstruction route; instead, it comprises a set of standardized operative modules, including total mesoesophageal excision and lymphadenectomy, gastric mobilization and abdominal lymphadenectomy, tubular conduit construction with retrosternal tunnel creation, cervical anastomosis, and ERAS‐based perioperative management [15]. Within this framework, our interest extends not only to whether cervical anastomotic leakage occurs and its incidence but also to its severity, clinical course, healing trajectory, and the maturation of system implementation over time.

Accordingly, we analyzed a consecutive cohort of 517 patients who underwent minimally invasive McKeown esophagectomy with retrosternal reconstruction within the SAFER surgical system to evaluate the incidence, severity, and outcomes of cervical AL. To strengthen comparative clinical interpretation, we additionally incorporated a posterior mediastinal reconstruction cohort comprising patients who developed cervical AL and compared leak severity, overall postoperative complication severity, mortality, healing rate, and time to closure between reconstruction routes. We further identified factors associated with delayed closure within the SAFER AL subcohort and characterized implementation maturity using RA‐CUSUM analysis.

## 2 Methods

### 2.1 Study Design and Patients

This was a single‐center retrospective cohort study including 517 consecutive patients with esophageal malignancy who underwent minimally invasive three‐incision McKeown esophagectomy under the SAFER surgical system at the Cancer Hospital, Chinese Academy of Medical Sciences, between 2023 and 2025. Inclusion criteria were: pathologically confirmed esophageal malignancy, clinical stage cT1‐4a/any N/M0, completion of minimally invasive McKeown esophagectomy with retrosternal reconstruction, and complete perioperative data. Tumors were staged according to the eighth edition AJCC/UICC classification [16]. Among the 517 patients, 50 developed cervical esophagogastric AL and comprised the leak subcohort for analyses of clinical course, time to closure, and determinants of delayed recovery.

To strengthen comparative interpretation of leak severity and healing outcomes, we additionally incorporated a historical same‐surgeon posterior mediastinal reconstruction comparator cohort. This cohort consisted of 37 patients who developed cervical AL after minimally invasive McKeown esophagectomy with posterior mediastinal reconstruction performed by the same lead surgeon before implementation of the SAFER surgical system. The comparator cohort was used specifically to compare the clinical consequences of cervical AL, including ECCG grade, Clavien–Dindo grade, mortality, healing rate, and time to closure. Because this historical comparator cohort included patients who had already developed cervical AL rather than a full denominator cohort of all posterior mediastinal reconstructions, it was not used to compare the incidence of AL between reconstruction routes.

### 2.2 The SAFER Surgical System

All operations were performed by the same dedicated surgical team in accordance with the SAFER low‐risk, high‐quality surgical system for esophageal cancer. SAFER is an optimized, pathway‐based surgical system centered on the retrosternal reconstruction route, developed by our team in collaboration with nine domestic centers, with the goal of reducing operative risk and improving overall quality of care. The core modules include: standardized total mesoesophageal resection of the thoracic esophagus with systematic lymphadenectomy while preserving the azygos arch and right bronchial artery; complete gastric mobilization with abdominal lymphadenectomy; creation of a tubular gastric conduit and construction of a retrosternal tunnel; cervical esophagogastric anastomosis; and an integrated enhanced recovery program. By meticulous anatomical separation and precise tunnel formation, SAFER effectively isolates the gastric conduit from the pleural and mediastinal spaces; together with standardized drainage strategies, this design aims to minimize intrathoracic contamination should an anastomotic leak occur. The conceptual goal of SAFER is not only to influence leak occurrence, but also to contain the clinical consequences of leakage through coordinated optimization of route selection, conduit orientation and tension, anatomical separation from the thoracic cavity, and standardized postoperative drainage and recovery management.

### 2.3 Data Collection

Clinical data were extracted from the institutional electronic medical record system, operative and anesthesia records, laboratory databases, and follow‐up records. Collected variables included demographic characteristics, body habitus, smoking and alcohol history, comorbidities, tumor location and stage, neoadjuvant treatment, anastomotic technique, perioperative laboratory parameters, and postoperative outcomes.

### 2.4 Outcome Measures

In the full cohort, the study assessed the incidence of cervical AL, Esophagectomy Complications Consensus Group (ECCG) severity grade, leak‐related mortality, and eventual closure, and further evaluated risk factors for AL occurrence. AL and its severity were defined according to the Esophagectomy Complications Consensus Group criteria: grade I, controlled with conservative treatment alone; grade II, requiring endoscopic or radiologic intervention; and grade III, requiring surgical reintervention [17].

Within the AL subcohort, the primary time‐to‐event outcome was defined as the interval from confirmed diagnosis of AL to documented leak closure or clinical healing. Clinical healing was defined as cessation or substantial reduction of local drainage, control of infection, and confirmation by imaging, endoscopy, or clinical assessment that no further leak‐directed intervention was required. The study specifically examined the association between POD1 albumin and healing trajectory by comparing time to closure between albumin‐defined groups. Low albumin was defined as a POD1 serum albumin level < 35 g/L [18], based on conventional clinical practice and prior literature.

In addition, a risk‐adjusted cumulative sum (RA‐CUSUM) curve based on AL occurrence was constructed to evaluate the dynamic evolution of outcomes and the progressive stabilization of SAFER system implementation over time [19].

### 2.5 Statistical Analysis

Continuous variables with a normal distribution were summarized as mean ± standard deviation and compared using Student's *t*‐test, whereas non‐normally distributed continuous variables were summarized as median (interquartile range) and compared using the Mann–Whitney *U* test. Categorical variables were compared using the chi‐square test or Fisher's exact test, as appropriate.

The posterior mediastinal cohort was an AL‐enriched comparator cohort without a full reconstruction‐route denominator; comparative analyzes were restricted to leak severity and healing outcomes rather than AL incidence.

Risk factors for leak occurrence in the full cohort were assessed using univariable and multivariable logistic regression. Within the leak subcohort, determinants of time to closure were assessed using Cox proportional hazards models, and results were reported as hazard ratios (HRs) with 95% confidence intervals. Kaplan–Meier curves were generated to depict cumulative closure probability, with an additional 30‐day landmark analysis.

Learning‐curve dynamics were analyzed using a risk‐adjusted cumulative sum (RA‐CUSUM) chart based on cervical anastomotic leakage occurrence. Expected leak risk for each case was estimated from a baseline logistic regression model fitted in the full cohort, including sex, age, body mass index, smoking status, diabetes mellitus, neoadjuvant therapy, and anastomotic method. Cases were ordered chronologically according to operative sequence, and the RA‐CUSUM was calculated as the cumulative sum of observed minus expected outcomes. The peak of the curve was interpreted as the transition from the earlier exploratory phase to the later, more stable phase. For descriptive presentation, observed leak rates were additionally summarized across sequential 50‐case blocks; the final 17 cases were treated as an incomplete terminal block and described separately. A two‐sided *p* value below 0.05 was considered statistically significant.

Statistical analyses were performed using R software (version 4.5.1) and IBM SPSS Statistics for Windows (version 26.0).

## 3 Results

### 3.1 Occurrence and Comparative Clinical Pattern of Cervical AL

Among 517 patients who underwent minimally invasive McKeown esophagectomy with retrosternal reconstruction within the SAFER surgical system, cervical AL occurred in 50 patients, yielding an overall AL rate of 9.7%. A historical same‐surgeon posterior mediastinal reconstruction cohort comprising 37 patients with cervical AL was included as a comparator for leak severity and healing outcomes. Baseline and perioperative characteristics of patients with cervical AL stratified by reconstruction route are shown in (Supplementary Table S1).

According to the ECCG classification, cervical AL in the SAFER group was predominantly grade I, with 36 grade I leaks (72.0%) and 14 grade II leaks (28.0%); no grade III leak was observed. In contrast, the PM cohort showed a higher‐severity ECCG distribution, with 4 grade I leaks (10.8%), 32 grade II leaks (86.5%), and 1 grade III leak (2.7%) (*p* < 0.001). According to the Clavien‐Dindo classification, the overall severity distribution was also more favorable in the SAFER group than in the PM cohort (*p* = 0.042). In the SAFER group, grade I, II, III, IV, and V events occurred in 10 (20.0%), 32 (64.0%), 5 (10.0%), 3 (6.0%), and 0 patients, respectively; the corresponding numbers in the PM cohort were 4 (10.8%), 16 (43.2%), 11 (29.7%), 5 (13.5%), and 1 (2.7%), respectively.

No in‐hospital, 30‐day, or 90‐day mortality occurred among the SAFER group with cervical AL, whereas one death occurred in the PM cohort for each mortality endpoint. All SAFER patients with cervical AL ultimately achieved healing, compared with 36 of 37 patients (97.3%) in the PM cohort. The median time from leak diagnosis to closure was shorter in the SAFER group than in the PM cohort (35 days [IQR, 27–50] vs. 46 days [IQR, 36–55], *p* = 0.011) (Table 1). Although most baseline and perioperative variables were comparable between the two leakage cohorts, tumor stage distribution differed significantly between groups.

**TABLE 1:** Clinical severity and healing course among patients with cervical anastomotic leakage stratified by reconstruction route.

Variable	RS group	PM group	*p* value
ECCG grade among patients with cervical AL, n/N (%)			< 0.001
Grade I	36/50 (72.0)	4/37 (10.8)	
Grade II	14/50 (28.0)	32/37 (86.5)	
Grade III	0/50 (0)	1/37 (2.7)	
Clavien–Dindo grade among patients with cervical AL, n/N (%)			0.042
Grade I	10/50 (20.0)	4/37 (10.8)	
Grade II	32/50 (64.0)	16/37 (43.2)	
Grade III	5/50 (10.0)	11/37 (29.7)	
Grade IV	3/50 (6.0)	5/37 (13.5)	
Grade V	0/50 (0)	1/37 (2.7)	
Mortality among patients with cervical AL, n/N (%)			
In‐hospital mortality	0/50 (0)	1/37 (2.7)	0.425
30‐day mortality	0/50 (0)	1/37 (2.7)	0.425
90‐day mortality	0/50 (0)	1/37 (2.7)	0.425
Healing outcome among patients with cervical AL			
Days from leak diagnosis to closure, median (IQR)	35 (27–50)	46 (36–55)	0.011
Eventual healing rate, n/N (%)	50/50 (100.0)	36/37 (97.3)	0.425

*Note:* ECCG grade reflects leak‐specific severity, whereas Clavien–Dindo grade reflects the overall severity of postoperative complications among patients who developed cervical AL.

Abbreviations: *AL, anastomotic leakage; ECCG, Esophagectomy Complications Consensus Group*.

### 3.2 Risk Factors for Cervical AL Occurrence in the SAFER Full Cohort

In univariable analysis, smoking (*p* = 0.023), diabetes mellitus (*p* = 0.013), neoadjuvant therapy (*p* = 0.002), and anastomotic method (hand‐sewn vs. stapled, *p* < 0.001) were associated with cervical AL occurrence. Variables with clinical relevance or a univariable *p* value < 0.10 were entered into the multivariable logistic regression model. After adjustment, diabetes mellitus remained independently associated with cervical AL occurrence (OR 2.91, 95% CI 1.39–6.10, *p* = 0.005), as did neoadjuvant therapy (OR 5.46, 95% CI 2.03–14.69, *p* < 0.001). Anastomotic method was also independently associated with AL occurrence, with hand‐sewn anastomosis showing a lower risk than stapled anastomosis (OR 0.03, 95% CI 0.01–0.09, *p* < 0.001). Smoking was no longer significant after adjustment (Table 2).

**TABLE 2:** Independent risk factors for cervical anastomotic leakage occurrence in the full cohort.

Variables	No. of patients (*N* = 517)	Univariable	Multivariable		
		*p* value	OR	95% CI	*p* value
Sex (male vs. female)	441/76	0.080	1.89	0.52–6.92	0.335
Age (years) (< 60 vs. ≥ 60)	94/423	0.135			
BMI (kg/m^2^) (< 22 vs. ≥ 22)	189/328	0.482			
Smoking (yes vs. no)	303/214	0.023	1.20	0.57–2.53	0.635
Alcohol use (yes vs. no)	284/233	0.647			
Diabetes mellitus (yes vs. no)	97/420	0.013	2.91	1.39–6.10	0.005
Hypertension (yes vs. no)	180/337	0.660			
Neoadjuvant therapy (yes vs. no)	338/179	0.002	5.46	2.03–14.69	< 0.001
Clinical stage (I vs. II vs. III vs. IV)	32/239/202/44	0.833			
Anastomotic method (Hand‐sewn vs. Stapled)	484/33	< 0.001	0.03	0.01–0.09	< 0.001

*Note:* Variables with clinical relevance or a univariable *p* value < 0.10 were entered into the multivariable logistic regression model. Cervical AL was defined according to ECCG criteria. Odds ratios are presented for the first category listed relative to the second category. An OR < 1 indicates lower odds of cervical AL occurrence for the first category relative to the second category.

Abbreviations: BMI, body mass index; CI, confidence interval; OR, odds ratio.

### 3.3 Predictors of Time to Closure in the SAFER AL Subcohort

Within the SAFER AL subcohort, univariable Cox regression identified smoking (*p* = 0.086), diabetes mellitus (*p* = 0.085), and low POD1 serum albumin (< 35 g/L; *p* < 0.001) as candidate factors associated with slower closure. In multivariable Cox regression, low POD1 albumin remained independently associated with a lower hazard of closure (HR 0.24, 95% CI 0.11–0.54, *p* < 0.001), indicating prolonged healing. Smoking was also independently associated with a lower closure hazard (HR 0.35, 95% CI 0.17–0.72, *p* = 0.004), whereas diabetes mellitus was not significant after adjustment (HR 1.31, 95% CI 0.62–2.74, *p* = 0.480) (Table 3).

**TABLE 3:** Predictors of time to closure among patients with cervical anastomotic leakage.

Variables	No. of patients (*N* = 50)	Univariable	Multivariable		
		*p* value	HR	95% CI	*p* value
Sex (male vs. female)	47/3	0.462			
Age (years) (≥ 60 vs. < 60)	37/13	0.572			
BMI (kg/m^2^) (< 22 vs. ≥ 22)	16/34	0.600			
Smoking (yes vs. no)	37/13	0.086	0.35	0.17–0.72	0.004
Alcohol use (yes vs. no)	29/21	0.611			
Diabetes mellitus (yes vs. no)	16/34	0.085	1.31	0.62–2.74	0.480
Hypertension (yes vs. no)	16/34	0.271			
Anastomosis method (Hand‐sewn vs. Stapled)	20/30	0.242			
Tumor staging (I vs. II vs. III vs. IV)	5/15/29/1	0.740			
Neoadjuvant therapy (yes vs. no)	43/7	0.382			
POD1 serum albumin (g/L) (< 35 vs. ≥ 35)	17/33	< 0.001	0.24	0.11–0.54	< 0.001

*Note:* Time to closure was defined as the interval from confirmed diagnosis of cervical AL to documented closure or clinical healing. POD1 albumin was entered into the Cox proportional hazards model as a categorical variable (< 35 vs. ≥ 35 g/L). HR < 1 indicates a lower instantaneous probability of achieving closure and therefore prolonged healing. The first category listed in parentheses was used as the reference category.

Abbreviations: CI, confidence interval; HR, hazard ratio; POD1, postoperative day 1.

### 3.4 POD1 Albumin and Healing Trajectory Among Patients With Cervical AL in the SAFER Group

**FIGURE 1:**
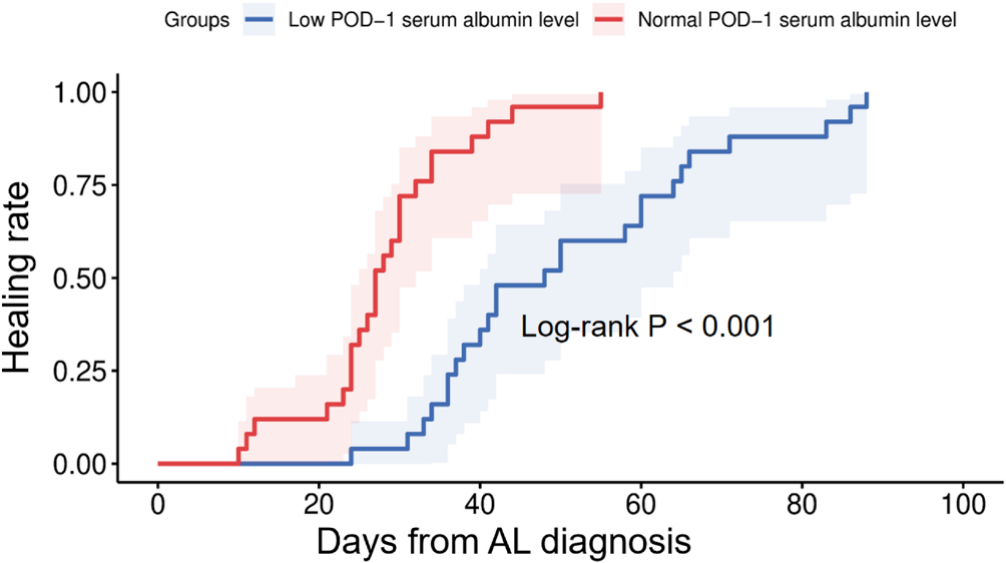
Kaplan–Meier curves for time to closure according to POD1 serum albumin level. Low POD1 serum albumin (< 35 g/L) was associated with a significantly delayed closure trajectory compared with normal POD1 serum albumin (≥ 35 g/L) (log‐rank *p* < 0.001).

Among the 50 patients with cervical AL, 17 had low POD1 serum albumin (< 35 g/L) and 33 had normal POD1 serum albumin (≥ 35 g/L). Baseline demographic and perioperative characteristics were generally comparable between the two groups. All patients in both groups ultimately achieved healing. However, patients with low POD1 albumin had a significantly longer time to closure than those with normal albumin levels (median 50.0 vs. 30.0 days, *p* < 0.001), as well as a longer postoperative hospital stay (median 68.0 vs. 52.0 days, *p* = 0.001). Kaplan–Meier analysis further demonstrated a significantly delayed closure trajectory in the low‐albumin group (Figure 1). In multivariable Cox regression, low POD1 albumin remained independently associated with a lower hazard of closure, indicating prolonged healing (Table 4). Sensitivity analysis using a 30‐day landmark approach yielded results consistent with the primary analysis. The low POD1 albumin group remained associated with a delayed closure trajectory both within the first 30 days and beyond the landmark time point (Figure S1).

**TABLE 4:** Clinical characteristics and healing outcomes of patients with cervical anastomotic leakage stratified by POD1 serum albumin.

Variables	Low albumin (< 35 g/L) (*n* = 17)	Normal albumin (≥ 35 g/L) (*n* = 33)	*p* value
Male, *n* (%)	15 (88.2%)	32 (97.0%)	0.264
Age (years), median (IQR)	64.0 (61.0–69.0)	62.0 (59.0–67.0)	0.250
BMI (kg/m^2^), mean ± SD	24.3 ± 2.4	22.9 ± 3.5	0.135
Smoking, *n* (%)	12 (70.6%)	25 (75.8%)	0.741
Alcohol use, *n* (%)	12 (70.6%)	17 (51.5%)	0.238
Diabetes mellitus, *n* (%)	5 (29.4%)	11 (33.3%)	0.778
Hypertension, *n* (%)	8 (47.1%)	8 (24.2%)	0.121
Neoadjuvant therapy regimen, *n* (%)			0.518
None	4 (23.5%)	3 (9.1%)	
nCT	4 (23.5%)	8 (24.2%)	
nCIT	4 (23.5%)	14 (42.4%)	
nCRT	2 (11.8%)	2 (6.1%)	
nCIT+nRT	3 (17.6%)	6 (18.2%)	
Clinical stage, *n* (%)			0.764
I	2 (11.8%)	3 (9.1%)	
II	4 (23.5%)	11 (33.3%)	
III	11 (64.7%)	18 (54.5%)	
IV	0 (0.0%)	1 (3.0%)	
Anastomotic method (Stapled), *n* (%)	10 (58.8%)	20 (60.6%)	1.000
Operation time (min), median (IQR)	300.0 (263.0–340.0)	268.0 (235.0–317.0)	0.231
Eventual healing, *n* (%)	17/17 (100.0)	33/33 (100.0)	1.000
Length of hospital stay (days), median (IQR)	68.0 (59.0–83.0)	52.0 (46.0–58.0)	0.001
Time to closure (days), median (IQR)	50.0 (40.0–65.0)	30.0 (24.0–36.0)	< 0.001

*Note:* Low albumin was defined as a POD1 serum albumin level < 35 g/L.

Abbreviations: BMI, body mass index; IQR, interquartile range; nCIT, neoadjuvant chemoimmunotherapy; nCIT+nRT, neoadjuvant chemoimmunotherapy plus radiotherapy; nCRT, neoadjuvant chemoradiotherapy; nCT, neoadjuvant chemotherapy; POD1, postoperative day 1; SD, standard deviation.

### 3.5 Maturation of SAFER System Implementation Assessed by RA‐CUSUM

**FIGURE 2:**
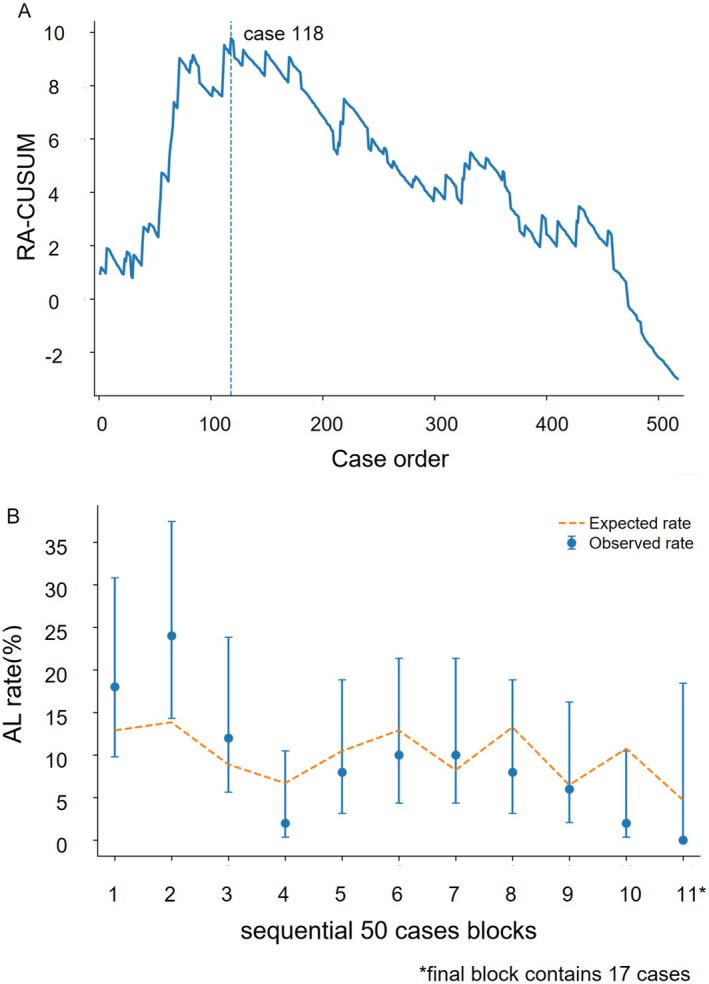
Maturation of SAFER system implementation assessed by cervical anastomotic leakage occurrence. A, RA‐CUSUM curve for cervical anastomotic leakage, showing an inflection point at approximately case 118. B, observed versus expected cervical anastomotic leakage rates across 10 complete sequential 50‐case blocks. The remaining 17 cases constituted an incomplete terminal block, in which no cervical anastomotic leakage occurred (0/17, 0.0%).

RA‐CUSUM analysis based on cervical AL occurrence showed an inflection point at approximately case 118, suggesting a transition from an early implementation phase to a relatively stable phase (Figure 2A). In the block‐based descriptive summary, the first 10 complete sequential 50‐case blocks showed observed cervical AL rates of 18.0%, 24.0%, 12.0%, 2.0%, 8.0%, 10.0%, 10.0%, 8.0%, 6.0%, and 2.0%, respectively. No cervical AL occurred in the remaining 17 cases (0/17, 0.0%). Overall, despite some fluctuation, the later phase showed a lower observed leakage burden than the earlier phase (Figure 2B).

## 4 Discussion

In our research, we incorporated a historical same‐surgeon posterior mediastinal reconstruction cohort to strengthen the comparative interpretation of cervical AL after minimally invasive McKeown esophagectomy. The principal finding was that cervical AL within the SAFER framework showed a less severe clinical pattern than AL after posterior mediastinal reconstruction. Although cervical AL still occurred in 9.7% of the SAFER group, most leaks were ECCG grade I, no ECCG grade III leak was observed, and all affected patients ultimately achieved healing. Compared with the PM cohort, the SAFER group had a more favorable ECCG distribution, a less severe Clavien‐Dindo distribution, no perioperative mortality, and a shorter median time to closure. These findings are consistent with the concept that SAFER surgical system may be most clinically relevant not simply as a strategy for eliminating leakage, but as a system for attenuating the consequences of leakage once it occurs.

Digestive tract reconstruction after esophagectomy is commonly carried out via the posterior mediastinal or retrosternal route. Additionally, practices regarding digestive tract reconstruction vary significantly between countries. In Japan, the retrosternal route is widely used (46.1%) [20], while in China, with only 6.2% of cases using the retrosternal route [9]. These data suggest that the retrosternal route remains underused in China and may have substantial room for further clinical adoption and technical standardization. The difference in leak severity between the SAFER and PM cohorts is anatomically and clinically plausible. In posterior mediastinal reconstruction, leakage from the cervical anastomosis or upper conduit may more readily communicate with the mediastinal or pleural spaces, thereby increasing the risk of deep contamination, pulmonary complications, systemic infection, and escalation of postoperative care. By contrast, the SAFER system uses a retrosternal‐centered reconstruction strategy in which the gastric conduit and cervical anastomosis are separated from the pleural cavity and posterior mediastinum. When leakage occurs, the infectious source is more likely to remain localized to the cervical or anterior route, allowing earlier recognition, local drainage, wound care, and nonoperative or minimally invasive management. This anatomical containment may explain why leaks in the SAFER group were more often low grade and less frequently associated with high Clavien‐Dindo severity.

Beginning in 2023, participating centers initiated a structured exploration of retrosternal‐centered reconstruction, progressively refining operative techniques and perioperative strategies. Through iterative multi‐center collaboration, this effort ultimately culminated in the formalization of the SAFER [15] system in 2025, characterized by modularized surgical steps, standardized tunnel creation, optimized conduit handling, and integrated recovery protocols. During the exploratory and establishment phases of this framework, more than 500 patients underwent retrosternal reconstruction under the SAFER conceptual structure. Importantly, the PM comparator cohort was derived from operations performed by the same lead surgeon before implementation of the SAFER system. This same‐surgeon design reduces inter‐operator variability and makes the comparison more clinically interpretable than a heterogeneous external cohort. In Supplementary Table S1, most baseline and perioperative variables among patients who developed cervical AL were generally comparable between groups, including sex, age, BMI, smoking history, alcohol use, diabetes mellitus, hypertension, neoadjuvant treatment regimen, and anastomotic method. However, tumor stage distribution differed between groups, and the historical nature of the PM cohort means that residual confounding and temporal changes in perioperative care cannot be fully excluded.

Also, the study concerns the trajectory of leak recovery. Although all 50 patients with AL ultimately achieved closure, healing time varied substantially. POD‐1 serum albumin emerged as an independent predictor of delayed healing in multivariable time‐to‐event analysis. Patients with low early postoperative albumin experienced a significantly reduced hazard of closure and prolonged recovery. Serum albumin, while not a direct measure of nutritional status alone, reflects a composite of physiological reserve, systemic inflammatory response, and perioperative stress. Early postoperative hypoalbuminaemia may therefore identify patients with diminished reparative capacity at a time when tissue healing is critically dependent on adequate protein synthesis and immune competence. From a clinical perspective, POD‐1 albumin is inexpensive, routinely available, and obtainable before overt clinical deterioration occurs. Within the SAFER framework, this finding has practical implications. Incorporating early albumin assessment into postoperative risk stratification may enable targeted nutritional optimization and intensified monitoring in patients at risk of prolonged leak recovery. Thus, SAFER may be conceptualized not only as an anatomical containment strategy but also as a dynamic governance model integrating structural risk isolation with early physiological vulnerability detection.

The RA‐CUSUM findings further suggest that the favorable clinical pattern observed within the SAFER cohort may reflect progressive system‐level maturation rather than a static route effect alone. We do not consider the observed inflection point to represent the “learning cost” of a single surgeon. Rather, it is more plausibly understood as a marker of implementation maturity at the system level. Beginning in 2023, participating centers initiated a structured exploration of retrosternal‐centered reconstruction, and the resulting technical refinements were subsequently formalized as the SAFER surgical system in 2025 [15]. The present cohort therefore spans both the exploratory and consolidation phases of this framework. In that context, the RA‐CUSUM pattern based on AL likely captures not only progressive technical familiarization but also the cumulative effects of modular standardization, team coordination, perioperative governance, and improved recognition and management of leakage. This interpretation is consistent with prior esophagectomy literature showing that outcome stabilization during the adoption of new techniques reflects more than operative dexterity alone [19, 21]. Moreover, systematic review data suggest that no single benchmark parameter has been universally accepted for minimally invasive esophagectomy learning‐curve assessment. Using cervical AL as the monitored endpoint is therefore conceptually appropriate in the present study, because AL is the complication that the SAFER system was explicitly designed to prevent or clinically contain. Accordingly, the RA‐CUSUM results are best viewed as evidence of progressive stabilization of SAFER implementation, rather than as a simple threshold defining when the procedure becomes “safe.”

Several limitations should be acknowledged. First, this was a retrospective study, and residual confounding cannot be excluded. Second, the posterior mediastinal comparator cohort was a historical same‐surgeon AL cohort rather than a contemporaneous full‐denominator control cohort. Although the same‐surgeon design reduced inter‐operator variability, temporal changes in perioperative management and residual selection bias could not be fully excluded. Therefore, the comparative analysis should be interpreted as a comparison of leak severity and healing outcomes among patients who developed cervical AL, rather than as evidence for route‐specific differences in AL incidence. Third, tumor stage distribution differed between the SAFER/RS and PM AL cohorts, which may have introduced additional imbalance despite the general comparability of other baseline and perioperative characteristics. Fourth, the number of leakage events was modest, which limits the stability of subgroup analyses and multivariable estimates. Finally, although the RA‐CUSUM analysis suggested progressive stabilization of SAFER implementation, the inflection point should not be overinterpreted as a universal minimum case volume for safe adoption. External multicenter validation using full‐denominator comparative cohorts will still be required to confirm whether the observed severity profile, the prognostic value of POD1 albumin, and the implementation trajectory of SAFER are reproducible across broader practice settings.

## 5 Conclusion

In conclusion, the SAFER system represents a structured, retrosternal‐centered surgical framework developed through multicenter collaboration and iterative refinement. In more than 500 patients treated during its exploratory and establishment phases, including 517 consecutive cases analyzed in this cohort, the incidence of cervical anastomotic leakage remained within the range reported in the literature; however, leakage events were predominantly low grade, clinically manageable, and ultimately healed in all affected patients. Compared with a historical same‐surgeon posterior mediastinal AL cohort, the SAFER/retrosternal group showed a more favorable leak‐specific and overall complication severity profile, with shorter time to closure. These findings suggest that a standardized retrosternal reconstruction strategy with the SAFER surgical system may help attenuate the severe clinical consequences traditionally associated with anastomotic leakage. Also, we find that POD1 serum albumin may further help identify patients at risk of delayed healing. Future full‐denominator comparative studies are warranted to validate these findings and further define the role of SAFER in esophageal cancer surgery.

## Author Contributions

Youyu Zhang, Weishi Wang, Yongjun Yu, Shenhai Wei, and Yong Li contributed to the conception and design of the study. Youyu Zhang, Weishi Wang, Yongjun Yu, Lixia Yin, Jintao Li, Weixin Liu, Xuefeng Zhang, Xingbo Zhang, Hong Yang, and Youyu Wang contributed to patient enrollment, clinical data collection, and data organization. Youyu Zhang, Weishi Wang, Yongjun Yu, and Yong Li performed the statistical analyses and interpreted the results. Youyu Zhang drafted the initial manuscript. Weishi Wang, Yongjun Yu, Shenhai Wei, and Yong Li contributed to critical revision of the manuscript for important intellectual content. Shenhai Wei and Yong Li supervised the study. All authors reviewed and approved the final version of the manuscript and agree to be accountable for all aspects of the work.

## Funding

This study was supported by the National Natural Science Foundation of China (No. 22478441), the CAMS Cancer Hospital Collaborative Fund (No. CFA202502024), and the National High Level Hospital Clinical Research Funding (No. 80102022501). The funders had no role in the study design, data collection, analysis or interpretation, manuscript preparation, or the decision to submit for publication.

## Ethics Statement

This study was approved by the Ethics Committee of Cancer Hospital, Chinese Academy of Medical Sciences (approval No. NCC2024C‐337), and the requirement for informed consent was waived due to the retrospective study design. All data were de‐identified prior to analysis and handled in accordance with applicable privacy and data protection regulations.

## Conflicts of Interest

The authors declare no conflicts of interest.

## Supporting information


**Figure S1:** Landmark analysis of time to closure according to POD1 serum albumin level.

**Table S1:** Supplementary Baseline and perioperative characteristics of patients with cervical AL stratified by reconstruction route.

## Data Availability

The datasets generated and analyzed during the current study are available from the corresponding author upon reasonable request. The data are not publicly available due to privacy and ethical restrictions.
