# Supplementary material for: Cervical Anastomotic Leakage After Minimally Invasive McKeown Esophagectomy in the SAFER Surgical System: Clinical Course and Predictors of Delayed Healing
Source: Thorac Cancer. 2026 Jun 10;17(11):e70314. doi: 10.1111/1759-7714.70314 (PMC13250757; doi:10.1111/1759-7714.70314)
Supplement: Supplementary file 1 — Figure S1: Landmark analysis of time to closure according to POD1 serum albumin level. [file TCA-17-e70314-s002.docx]

**Figure S1. Landmark analysis of time to closure according to POD1 serum albumin level.**


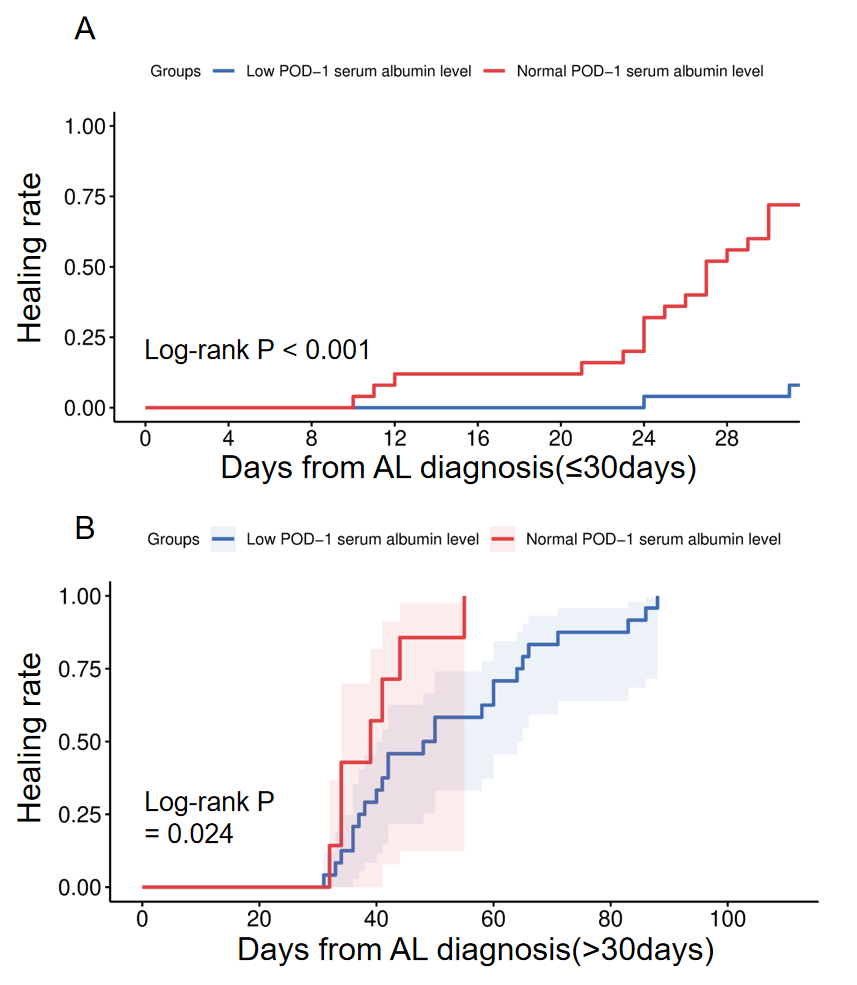


*Abbreviations: Thirty-day landmark analysis of time to closure according to POD1 serum albumin level. A: Closure trajectory within 30 days after AL diagnosis; B: Closure trajectory beyond 30 days among patients remaining unhealed at the landmark time point; Curves were compared using the log-rank test.*
