# Supplementary material for: Cervical Anastomotic Leakage After Minimally Invasive McKeown Esophagectomy in the SAFER Surgical System: Clinical Course and Predictors of Delayed Healing
Source: Thorac Cancer. 2026 Jun 10;17(11):e70314. doi: 10.1111/1759-7714.70314 (PMC13250757; doi:10.1111/1759-7714.70314)
Supplement: Supplementary file 2 — Table S1: Supplementary Baseline and perioperative characteristics of patients with cervical AL stratified by reconstruction route. [file TCA-17-e70314-s001.docx]

**Supplementary Table S1. Baseline and perioperative characteristics of patients with cervical AL stratified by reconstruction route**

| Variable | RS group (n=50) | PM group (n=37) | P value |
| --- | --- | --- | --- |
| Sex, n (%) |  |  | 0.277 |
| Male | 47/50 (94.0) | 32/37 (86.5) |  |
| Female | 3/50 (6.0) | 5/37 (13.5) |  |
| Age (years), median (IQR) | 63.0 (59.0–67.0) | 62.7 (58.1–69.5) | 0.904 |
| BMI (kg/m²), mean ± SD | 23.4 ± 3.2 | 23.5 ± 2.5 | 0.836 |
| Smoking history, n (%) | 37/50 (74.0) | 26/37 (70.3) | 0.809 |
| Alcohol use, n (%) | 29/50 (58.0) | 24/37 (64.9) | 0.657 |
| Diabetes mellitus, n (%) | 16/50 (32.0) | 8/37 (21.6) | 0.338 |
| Hypertension, n (%) | 16/50 (32.0) | 15/37 (40.5) | 0.499 |
| Neoadjuvant therapy regimen, n (%) |  |  | 0.746 |
| None | 7/50 (14.0) | 7/37 (18.9) |  |
| nCT | 12/50 (24.0) | 9/37 (24.3) |  |
| nCIT | 18/50 (36.0) | 15/37 (40.5) |  |
| nCRT | 4/50 (8.0) | 3/37 (8.1) |  |
| nCIT+nRT | 9/50 (18.0) | 3/37 (8.1) |  |
| Tumor stage, n (%) |  |  | 0.013 |
| Stage I | 5/50 (10.0) | 11/37 (29.7) |  |
| Stage II | 15/50 (30.0) | 15/37 (40.5) |  |
| Stage III | 29/50 (58.0) | 9/37 (24.3) |  |
| Stage IV | 1/50 (2.0) | 1/37 (2.7) |  |
| Unknown/missing | 0/50 (0.0) | 1/37 (2.7) |  |
| Anastomotic method, n (%) |  |  | 0.104 |
| Hand-sewn | 20/50 (40.0) | 8/37 (21.6) |  |
| Stapled | 30/50 (60.0) | 29/37 (78.4) |  |

Abbreviations: AL, anastomotic leakage; BMI, body mass index; IQR, interquartile range; SD, standard deviation; RS, retrosternal reconstruction; PM, posterior mediastinal reconstruction; nCT, neoadjuvant chemotherapy; nCIT, neoadjuvant chemoimmunotherapy; nCRT, neoadjuvant chemoradiotherapy; nCIT+nRT, neoadjuvant chemoimmunotherapy plus radiotherapy. P values for sex, neoadjuvant regimen, tumor stage, and anastomotic method represent overall distribution comparisons.
